# MIBG Therapy for Neuroblastoma: Precision Achieved With Dosimetry, and Concern for False Responders

**DOI:** 10.3389/fmed.2020.00173

**Published:** 2020-05-28

**Authors:** Pedro M. Rubio, Victor Galán, Sonia Rodado, Diego Plaza, Leopoldo Martínez

**Affiliations:** ^1^Pediatric Hemato-Oncology Department, Hospital Universitario La Paz, Madrid, Spain; ^2^Investigación Traslacional en Cáncer Infantil, Trasplante Hematopoyético y Terapia Celular, Instituto de Investigación Hospital Universitario La Paz (IdiPAZ), Madrid, Spain; ^3^Nuclear Medicine Department, Hospital Universitario La Paz, Madrid, Spain; ^4^Pediatric Surgery Department, Hospital Universitario La Paz, Madrid, Spain; ^5^Network for Maternal and Children Health SAMID (RD16/0022/0006), Instituto de Salud Carlos III, Madrid, Spain

**Keywords:** dosimetry, mIBG, neuroblastoma, mIBG therapy, false responder

## Abstract

Neuroblastoma causes 15% of cancer mortality in children. High risk neuroblastoma has poor prognosis, with high relapse rate and mortality despite multimodal treatment. 123-I-meta-iodo-benzyl-guanidine (mIBG) scintigraphy is one of the current standard diagnostic procedures in neuroblastoma. mIBG can also be used therapeutically, labeled with 131-I, as a radiopharmaceutical agent, delivering targeted radiotherapy to tumoral sites. But published data of this strategy show heterogeneous results. One concern is that in most reports the infused activity is only based in body-weight, which could lead to infra or over-treatment, depending on inter-patient variability in radiation absorption. Activity adjustment by whole-body dosimetry can be used to homogeneize the treatment. Also, mIBG avid tumors may lose avidness along the treatment. As mIBG is used both for treatment and response evaluation, this could result in undetected progressions in patients with apparent complete response. We present a retrospective single-center review of neuroblastoma patients who received therapeutic 131-I-mIBG, focusing on cases with dosimetry-adjusted activity. Dosimetry allowed for a more precise delivery of radiation, reducing 81.1% of deviation from absorption target of 4 Gray (Gy), from 23.4% (±0.936 Gy) to 4.4% (± 0.176 Gy). Patients who showed partial or complete response had better and longer survival. Relapse/progression in non-responders was an early event (within 3 months from treatment). We also present one case of progression with apparent complete response due to loss of mIBG avidness, detected in our series.

## Introduction

Neuroblastoma is a neoplasia derived from the autonomic nervous system precursors. It represents 10% of childhood cancer diagnoses and 15% of its mortality, and is the most frequent malignant tumor below 1 year of age. Approximately 40% of patients present with high-risk neuroblastoma (HRN), which arises in older children, harbors several unfavorable genetic alterations (MYCN amplification, structural chromosome aberrations with gains and losses especially in 1p, 11q, and 17q), and is usually metastatic, mainly to bone and bone marrow (1, 2).

Classical HRN therapy combines chemotherapy–both conventional, and high-dose with autologous stem cell rescue-, surgery, radiotherapy, and isotretinoin maintenance. Recently, immunotherapy has been incorporated to first line HRN treatment, after a pivotal report by Yu et al., which showed a 20% survival advantage for patients receiving anti-disialoganglioside (GD2) antibody therapy (3, 4).

Despite being a prolonged, intensive and toxic treatment, even the latest data of long-term survival (64% overall survival 5 years after diagnosis) are poor in comparison with other pediatric tumors (83.7% in the Surveillance, Epidemiology, and End Results [SEER] Program 2019 report) (4, 5). Relapse rate remains high at 30–40%, with very low relapse survival (6–8). New approaches are needed for non-responders and relapse patients.

Meta-iodo-benzyl-guanidine (mIBG) is a norepinephrine analog –a false neurotransmitter. Its uptake by cathecolaminergic cells makes it an ideal marker for neuroblastoma. mIBG scintigraphy has been for decades a standard procedure for extension evaluation and metastatic response assessment (9, 10).

Since its approval by the FDA in 2008, I-123 is the isotope of choice for mIBG studies (11). The I-131 isotope is less used for diagnosis, due to its poorer photon flux (which results in less quality imaging), and higher toxicity because of its longer half-life (8 days vs 13 h of I-123) and higher energy radiation (I-131 is a beta-emitter) (12). Precisely these two latter features make it a useful therapeutic agent. Uptake of mIBG by the Nor Epinephrine Transporter (NET), present in most neuroblastomas, ensures that the isotopic activity is delivered to tumoral avid sites (13).

Although there is abundant literature on mIBG therapy, heterogeneity of patients and schedules makes it difficult to assess its true effectiveness (14, 15). A study from the New Approaches for Neuroblastoma Therapy (NANT) Consortium identified 444 megabecquerels per kilogram (MBq/kg) as the maximum tolerated dose, which subsequently became standard (16). The use of a fixed activity based only on patient's weight does not account for variability both of uptake in tumors/normal organs and of whole-body retention, and could lead to under or over-treatment of patients. To amend this, whole-body dosimetry (WBD) can be used to adjust I-131 activity administered to an absorption target (17–19).

In this report, we review retrospectively our experience with mIBG therapy in neuroblastoma, focusing on the impact of WBD on the precision delivery of radiation.

We also present a case of false complete response due to loss of mIBG avidness in a previously positive patient.

Approximately 10% neuroblastomas do not express NET, and are mIBG-negative from diagnosis (20–22). Also, mIBG-positive neuroblastomas might stop expressing NET and lose mIBG avidness. This is a serious concern, as undetected progressions might occur in patients with apparent complete response in the mIBG scan. We have found one such case in our series. We have failed to find any case in the literature.

## Materials and Methods

This is a single-center, retrospective, descriptive review of mIBG therapy in neuroblastoma patients. Inclusion criteria were: diagnosis of neuroblastic tumor, diagnosed and/or treated at any point in our Hospital, and having received 131-I-mIBG treatment regardless of disease status. Demographic, clinical, toxicity, and outcome data were collected from patients' records. Images were collected from the hospital database and anonymized.

Software used for data collection included Microsoft Office 2003 for Windows (Access and Excel). IBM SPSS Statistics for Windows (IBM Corp. Released 2017. Armonk, NY: IBM Corp) was used for the statistical analysis. Fisher's exact test was used for group comparison.

Tumor staging was based on the revised International Neuroblastoma Staging System (INSS), and response assessment adapted from the International Neuroblastoma Response Classification (INRC) (23). Complete Response (CR) when there was no detectable tumor; Partial Response (PR) when there was a reduction in tumor burden; Stable Disease (SD) when there was no change in tumor burden; and Progressive Disease (PD) when a tumor volume increase, or appearance of new lesions. Treatments which resulted in CR or PR were considered ‘responders’.

### Survival, Progression and Response Assessment

Survival was calculated for all patients from the first day of the first mIBG therapy. As some patients received more than one mIBG therapy, response and time to progression are evaluated for each treatment. For any given patient, only treatments administered in different settings (i.e., first-line and relapse, with a treatment-free interval) were considered.

### Fixed and Dosimetry Groups

Patients treated with a fixed 131-I-mIBG activity, based only on body weight, formed the Fixed-dose Group (FG). The Dosimetry Group (DG) included patients with 131-I-mIBG treatment divided in two infusions. The first infusion was weight-based (444 MBq/kg) and the second adjusted by WBD, with the aim of reaching an absorbed dose of 4 Gy with the full treatment.

### Whole Body Dosimetry

WBD was calculated according to Buckley et al. (24). Measurements were taken with the patient in a reproducible position (lateral decubitus) from front and back, at 1 meter, with a verified Geiger dosimeter, in micro Sieverts per hour (μSv/h). Measurements took place every 2 h the first day and every 4 to 6 h the following days. We used a factor MBq/μSv/h obtained immediately after administration of the activity to the patient without emptying the bladder.

The results were represented in a time-activity curve, adjusted by a biexponential, and integrated to obtain the accumulated activity $\overset{-}{\text{A}}$.

The result in Gy was obtained according to the Committee on Medical Internal Radiation Dose (MIRD) methodology:

- Mean absorbed dose: D _WB < −WB_ = $\overset{-}{\text{A}}$. S _WB < −WB_.
- Where S _WB < −WB_ = 1.34 x 10 ^−4^ mp ^−921^ Gy. MBq ^−1^. h ^−1^
- And mp = patient's mass in kg.

## Results

From September 1992 to January 2019, 32 mIBG treatments were administered to 29 patients (15 males, 14 females), representing 11.3% of the 225 neuroblastomas attended in our institution during that period.

Most patients (22/29: 75.8%) had metastatic disease: 20 were stage 4 (metastatic including bone, lung and/or central nervous system) and 2 stage 4s (children below 18 months of age, with limited metastases, excluding stage 4 sites: this group has a more favorable prognosis). Most frequent sites of metastasis were bone (20/29: 69%) and bone marrow (17/29: 58.6%). Primary tumor location was predominantly the abdomen (24/29, 82.8%).

Almost half of the patients (14/29, 48.3%) had been treated within the 1990's Spanish Pediatric Oncology Society (SEOP) SOP protocols. Of the remaining 15 patients, 9 received modern high-risk protocols (International Society of Pediatric Oncology SIOPEN HR protocol, German Pediatric Oncology Group GPOH NB2004), 5 low-risk or infant protocols (SIOPEN EUNS and INES), and 1 patient had only undergone surgical resection prior to mIBG therapy. Overall, 79% of the patients had been classified as HRN at diagnosis.

Median age at mIBG therapy was 73.5 months, with an interquartile range (IQR) of 44.5–95.5 months.

Only 6 patients received mIBG as a first-line [FL] treatment: 5 within the SOP protocols, and 1 SIOPEN HR patient who received it off-protocol by parents' choice. All patients had been classified as stage 4, although in our review we found that 1 was overstaged and was in fact a 4s.

The remaining 26 treatments were administered in a relapse (REL: 17/26) or refractory (RF: 9/26) setting.

Three patients received mIBG therapy more than once, with a treatment-free interval in between (patient #4 in first-line and relapse, and patients #10 and #15 in successive relapses).

The FG included 24 patients and 27 treatments; the DG included 5 patients and 5 treatments. Demographics and clinical data are summarized in Table 1.

**Table 1:** Patients characteristics.

	**ALL (*n* = 29)**	**DG (*n* = 5)**	**FG (*n* = 24)**	***p***
**Sex**				0.186
Male	15 (51.7%)	4 (80%)	11 (45.8%)	
Female	14 (48.3%)	1 (20%)	13 (54.2%)	
**Age in months:**	**Median (IQR)**	**Median (IQR)**	**Median (IQR)**	
At neuroblastoma diagnosis	42 (29.5–55)	45 (36–78.5)	41.5 (26–50)	0.434
At MIBG treatment	73.5 (44.5–95.5)	107 (63.5–124)	71 (42–89)	0.219
**Primary tumor site**				0.170
Cervical	3 (10.3%)	2 (40%)	1 (4.2%)	
Thoracic	1 (3.4%)	-	1 (4.2%)	
Abdominal-Pelvic	24 (82.8%)	3 (60%)	21 (87.5%)	
*Adrenal*	*19 (62%)*	*3 (60%)*	*15 (62.5%)*	
No primary	1 (3.4%)	-	1 (4.2%)	
**Side**				0.511
Left	8 (27.6%)	1 (20%)	7 (29.2%)	
Right	15 (51.7%)	4 (80%)	11 (45.8%)	
Bilateral	3 (10.3%)	-	3 (12.5%)	
Missing	*3 (10.3%)*	-	3 (12.5%)	
**Stage (INSS)**				0.865
2a	1 (3.4%)	-	1 (4.2%)	
3	6 (20.6%)	1 (20%)	5 (20.8%)	
4s	2 (6.9%)	-	2 (8,3%)	
4	20 (69%)	4 (80%)	16 (66.7%)	
**MYC-N Status**				0.515
Amplified	2 (6.9%)	1 (20%)	1 (4.2%)	
Non amplified	15 (51.7%)	4 (80%)	11 (45.8%)	
Unknown	*12 (41.4%)*	-	*12 (50%)*	
**Location of metastases:**				
Bone	20 (69%)	4 (80%)	16 (66. 7%)	0.498
Bone marrow	17 (58.6%)	3 (60%)	14 (58.3%)	0.671
Lymph nodes	10 (34.5%)	2 (40%)	8 (33.3%)	0.576
Liver	4 (13.8%)	-	4 (16.7%)	
CNS	1 (3.4%)	-	1 (4.2%)	
Skin	1 (3.4%)	-	1 (4.2%)	
**First line treatment**				0.068
SOP90s	14 (48.3%)	-	14 (58.3%)	
SIOPEN HRNBL	8 (27.6%)	3 (60%)	5 (20.8%)	
GPOH NB2004	1 (3.4%)	1 (20%)	-	
EUNS	3 (10.3%)	1 (20%)	2 (8.3%)	
INES99	2 (6.9%)	-	2 (8.3%)	
SURGERY	1 (3.4%)	-	1 (4.2%)	

*DG, dosimetry group; FG, “fixed-activity” group; IQR, InterQuartile Range (25–75%); INSS, International Neuroblastoma Staging System (see note below); CNS, Central Nervous System; SOP90s, Spanish Sociedad de Oncología Pediátrica protocols (from 1990 to 1998); GPOH NB2004, Gesellschaft für pädiatrische Onkologie und Hämatologie high-risk neuroblastoma protocol; SIOPEN, European SIOP Neuroblastoma group; HRNBL, High risk neuroblastoma protocol; INES99, Infant Neuroblastoma Study; EUNS, European Unresectable Neuroblastoma Study*.

*INSS staging (summarized): (1) Localized tumor with complete gross excision. (2a) Localized tumor with incomplete gross excision, lymph nodes negative. (2b) Localized tumor with ipsilateral lymph nodes positive. (3) Unresectable unilateral tumor infiltrating across the midline, or contralateral regional lymph node involvement; or midline tumor with bilateral extension (4) Dissemination to distant lymph nodes, bone, bone marrow, liver, skin, and/or other organs, except as defined for stage 4S. (4s) Localized primary tumor with dissemination limited to skin, liver, and/or bone marrow, limited to infants younger than 12 months*.

Response to mIBG therapy after 32 treatments was: 6 CR (18.8%), 9 PR (28.1%), 5 SD (15.6%), and 12 PD (37.5%). Overall Response Rate (CR+PR) was 46.9%. According to treatment setting, ORR was 83.3% in FL, 41.2% in REL, and 33.3% in RF.

Progression followed after 81.3% of treatments (26/32). It was similar in all treatment settings (FL: 83.3%; REL: 82.4%; RF: 77.8%). In the CR + PR group, progression rate was 60%, while all SD and PD cases progressed. Progression rate in the DG was 60%, and in the FG it was 85.2%.

Time to progression was not available for 3 treatments. Median progression-free interval (PFI) for 29 evaluable treatments was 7 months (IQR 1-15). Median PFI and IQR for responders (CR + PR) was 15 (8–102) months, and for non-responders (NR: SD + PD) 1 (1–3) months. Early progressions were rare in responders (0 at 3 months, 13.3% at 6 months), while 85.7% of non-responders progressed in the first 3 months. In the CR group there was 1 relapse in the first 9 months after mIBG treatment.

After a median follow-up period of 6 months (IQR 2.5–47), overall survival (OS) was 37.9% and event-free survival (EFS) 20.7%.

Most deaths took place early in the follow-up (50% in the first 4 months, and 66.7% in 6 months). Median follow-up for survivors was 51 months (IQR 1–124), and for patients who stayed in CR was 90.5 months (IQR 41.5–124.25).

Survival in the DG was 40% (OS and EFS), and in the FG 37.5% (OS) and 16.7% (EFS). Outcome data are summarized in Table 2.

**Table 2:** Outcomes of MIBG therapy.

	ALL^*^	**DG**	**FG**	**DG Vs FG (p)**
**Responders (CR+PR)**	15/32 (46.9%)	2/5 (40%)	13/27 (48.1%)	0.737
CR	5 (15.6%)	1 (20%)	5 (18.5%)	
PR	10 (31.3%)	1 (20%)	8 (29.6%)	
SD	5 (15.6%)	-	5 (18.5%)	
PD	12 (37.5%)	3 (60%)	9 (33.3%)	
**OS**	37.9% (11/29)	40% (2/5)	37.5% (9/24)	0.917
Responders (CR+PR)	46.7% (7/15)	100% (2/2)	38.5% (5/13)	0.200
NR (SD+PD)	28.6% (4/14)	0% (3/3)	36.4% (4/11)	0.33
Responders Vs NR (p)	0.268	0.100	0.625	
**EFS**	20.7% (6/29)	40% (2/5)	16.7% (4/24)	0.241
Responders (CR+PR)	40% (6/15)	100% (2/2)	30.8% (4/13)	0.143
NR (SD+PD)	0% (0/14)	0% (3/3)	0% (0/11)	NA
Responders Vs NR (p)	**0.011**	0.100	0.067	
	Median (IQR)	Median (IQR)	Median (IQR)	
**PFI (months)**	7 (1–15)	2 (1–32)	7.5 (1–15)	0.369
Responders (CR+PR)	15 (8–102)	32 (13–NA)	15 (8–113)	0.742
NR (SD+PD)	1 (1–3)	1 (1–NA)	1 (1–3)	0.169
Responders Vs NR (p)	**0.005**	0.119	**0.015**	

* *32 treatments were considered for response analysis and 29 patients for survival*.

*Comparisons have been made between responders and non-responders (NR), shown in the rows below the groups, and between DG and FG, shown in the last column. Statistically significant differences are highlighted in bold text*.

*No statistically significant differences could be found between DG and FG in response, OS, EFS nor PFI. Responders had better EFS (p = 0.011) and longer PFI (p = 0.005 for the whole cohort, p = 0.015 in the FG)*.

*DG: dosimetry group; FG: “fixed-activity” group; CR: complete response; PR: partial response; SD: stable disease; PD: progressive disease; NR: non-responders (SD + PD); OS: overall survival; EFS: event-free survival; PFI: progression-free interval; IQR: interquartile range*.

Toxicity data in FG patients was too scarcely and heterogeneously reported to be useful for analysis; in DG patients no relevant toxicities beyond hematological grade 4 were reported.

### Dosimetry Data

DG patients received two I-131 infusions separated by 14 days. After the first (444 MBq/kg), WBD was performed to adjust the second infusion to a target of 4 Gy absorption for the whole treatment.

Dosimetry showed an average deviation of 23.4% from the target absorbed dose (2 Gy) after the first infusion. All patients required adjustment of the second mIBG activity, which reduced deviation by a factor of 81.1 to 4.4% from the 4 Gy absorption target (0.936 to 0.176 Gy). Dosimetry data are summarized in Table 3.

**Table 3:** Dosimetry group—activity adjustment and dispersion control.

	**Case 1**	**Case 2**	**Case 3**	**Case 4**	**Case 5**	**All**
First infusion, activity (MBq/kg)	444	444	444	444	444	
First infusion, absorbed radiation (Gy)	2.03	1.32	2.16	3.09	2.38	
% of target absorption	101.5%	66%	108%	154.5%	119%	
Deviation before adjustment^**^	*1.5%*	*34%*	*8%*	*54.5%*	*19%*	*23.4%*
Second infusion, activity (MBq/kg)	450	713	334	191	360	
Absorbed radiation (Gy)	1.99	2.42	1.96	0.95	1.18	
*Total absorbed radiation (Gy)*	*4.02*	*3.74*	*4.12*	*4.04*	*3.56*	
% of target absorption	100.5%	93.5%	103%	101%	89%	
*Deviation after adjustment*	*0.5%*	*6.5%*	*3%*	*1%*	*11%*	*4.4%*

*Target absorbed radiation was 2 Gy per infusion, 4 Gy total. After the first infusion, calculated absorbed dose showed an average deviation of 23.4%. Correction of the second activity reduced 81.1% of this deviation, which was 4.4% for the whole treatment. MBq/kg, megaBecquerel per kilogram; Gy, gray. Second activity is shown per weight to illustrate better the difference from the first infusion*.

### Loss of mIBG Avidness Mimicking CR

**Figure 1:**
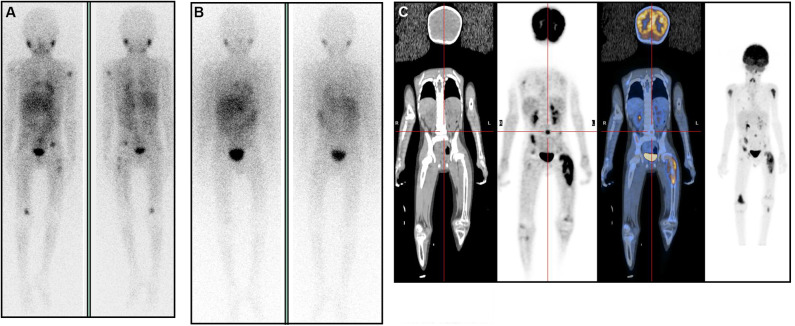
False complete response after 131-I-mIBG therapy: **(A,B)** 123-I-mIBG scans. **(C)** 18-F-FDG-PET-CT scan. **(A)** positive scan previous to 131-I-mIBG therapy, with multiple spots. **(B)** negative post-mIBG therapy scan (Complete Response). **(C)** as response was much better than expected, a post-therapy PET-CT scan was requested, showing non-mIBG-avid Progressive Disease.

One patient from the DG was diagnosed in 2016 of a multifocal relapse of a stage 4, MYCN amplified neuroblastoma. Relapse treatment with TVD chemotherapy (topotecan, vincristine, and doxorubicin) was started. After 4 cycles response was SD, and 131-I-mIBG was administered. Post-treatment mIBG scan showed a striking CR. As the response was unexpected, an 18F-FDG-PET-CT scan was performed, showing PD (Figure 1). An ALK mutation was detected in the relapse sample, so crizotinib was proposed, but rapid progression followed and he died 3 months after mIBG therapy.

## Discussion

We present a small retrospective review of mIBG therapy with the remarkable finding of loss of mIBG avidness in one patient. Furthermore, dosimetry data in our cohort showed important differences between planned and calculated radiation absorption, which led to activity adjustment in all patients.

In an effort to standardize mIBG therapy, dosimetry is essential. Gaze et al proposed, over a decade ago, a WBD-based combination of mIBG with topotecan chemotherapy as radiosensitizer (18). This proposal has been incorporated to SIOPEN strategy for refractory neuroblastoma in the VERITAS trial (20). In our DG patients, dosimetry showed important differences between the planned and actual radiation absorption, which could be reduced below 5% with the two-infusion strategy. In a recent Spanish report of 10 patients, 9 required activity adjustment, ranging from 4.5 to 140% of the theoretical dose (25).

Loss of mIBG avidness has only rarely been reported (21, 22). Aside from the case reported in this review, we have identified a mIBG-negative relapse in another patient from our center, who was not included in this paper as he did not receive mIBG therapy. We suspect this could have also happened in older patients, before PET-CT was available. One patient in our series, treated in May 2000, had a reported CR. However, an early metastatic relapse was diagnosed by MRI, which led to his death 5 months after mIBG treatment. This was the only case of short-term progression in the CR group, and this pattern is more consistent with a false negative mIBG. To prevent undetected progression due to loss of mIBG avidness, PET-CT scanning prior and post 131-I-mIBG therapy should be a standard procedure.

As mIBG therapy has been until now a rescue treatment for a rare disease like neuroblastoma, it is difficult to extract outcome conclusions in a single-center study. Patients in our sample are extended over a long period (27 years) and heterogeneity is abundant in treatment regimens and patient conditions. Even so, our findings are comparable with the literature.

Recently, Wilson et al summarized the results of over 30 papers comprising almost 1000 patients. Overall response rate was 30%, which was similar to Zhou's report in 2015. Both reports focused on relapse patients (14, 26). In those studies, FL treatments yielded ORRs of 56–66% (15). Our data are slightly better, with an ORR of 46.9% in the whole cohort, and 83.3% in the FL group. We found correlation between response and EFS (*p* = 0.011). Responders had also a longer PFI (*p* = 0.005), being rare the early events in this group.

We could not statistically prove differences in outcome between treatment settings (FL, REL and RF). Response rate seemed better in FL patients (*p* = 0.062), while no difference in both OS and EFS could be found. Larger series report poorer prognosis for refractory patients (26). Also, no difference in response nor survival could be shown between DG and FG, possibly due to small sample size and short follow up in the DG.

Our report presents several weak points. Sample size is always an issue in unicentric pediatric cancer reports, especially when researching a non-standard second-line treatment. This also results in heterogeneity, with patients treated in different eras with diverse regimes.

Short median follow-up is influenced by the frequent and early events in these patients: thus, it is significantly longer in survivors (51 months, *p* = 0.006) and responders (15 months, *p* = 0.005).

We hope to take advantage of the progressive standardization of treatments and monitoring of dosing and response: VERITAS protocol is expected to bring in the future much more abundant data on the role of directed radiotherapy in neuroblastoma.

Overall, our findings are consistent with the literature and show that mIBG is a feasible therapy for neuroblastoma. Dosimetry seems to be essential to deliver a precise amount of targeted radiotherapy for these patients. Every CR should be confirmed by other method (i.e., PET scan) to detect avidness loss. Incorporation of mIBG to first line treatment in the future SIOPEN HRNBL-2 protocol, and in the refractory setting in the ongoing VERITAS study are expected to provide richer data for this therapy, and hopefully an improvement in survival for patients with aggressive neuroblastoma.

## Data Availability Statement

The datasets generated for this study are available on request to the corresponding author.

## Ethics Statement

Patients in this study have been treated mostly under SOP (Spanish Pediatric Oncology Society) and SIOPEN (International Society of Pediatric Oncology Neuroblastoma Group) protocols, which all have EC-approval. Patients outside study protocols (as relapses or referral from other centers) provided additional informed consent for procedures in our center. As the retrospective study described is within the bounds of regular clinical practice and written informed consent to participate in this study was provided by the participants' legal guardian/next of kin, ethical review and approval was not required for the study on human participants in accordance with the local legislation and national guidelines.

## Author Contributions

PR, VG, and LM contributed to the conception and design of the paper. PR, VG, and DP created and fulfilled the database. PR and VG performed the statistical analysis and wrote the first draft of the manuscript. SR obtained and calculated dosimetry data. LM coordinated the composition of the manuscript. All authors wrote sections of the manuscript. This paper is part of a PhD project. As such, it should be explicitly stated that, although PR is the first and corresponding author, VG should be considered first author as well, as they had the same responsibilities in the elaboration of this manuscript. All authors contributed to manuscript revision, read and approved the submitted version.

## Conflict of Interest

The authors declare that the research was conducted in the absence of any commercial or financial relationships that could be construed as a potential conflict of interest.
